# Each protomer of a dimeric YidC functions as a single membrane insertase

**DOI:** 10.1038/s41598-017-18830-9

**Published:** 2018-01-12

**Authors:** Dirk Spann, Eva Pross, Yuanyuan Chen, Ross E. Dalbey, Andreas Kuhn

**Affiliations:** 1 Institute of Microbiology and Molecular Biology, University of Hohenheim, 70599 Stuttgart, Germany; 2 Department of Chemistry and Biochemistry, Ohio State University, Columbus OH, 43210 USA

## Abstract

The membrane insertase YidC catalyzes the entrance of newly synthesized proteins into the lipid bilayer. As an integral membrane protein itself, YidC can be found as a monomer, a dimer or also as a member of the holotranslocase SecYEGDF-YajC-YidC. To investigate whether the dimeric YidC is functional and whether two copies cooperate to insert a single substrate, we constructed a fusion protein where two copies of YidC are connected by a short linker peptide. The 120 kDa protein is stable and functional as it supports the membrane insertion of the M13 procoat protein, the C-tailed protein SciP and the fusion protein Pf3-Lep. Mutations that inhibit either protomer do not inactivate the insertase and rather keep it functional. When both protomers are defective, the substrate proteins accumulate in the cytoplasm. This suggests that the dimeric YidC operates as two insertases. Consistent with this, we show that the dimeric YidC can bind two substrate proteins simultaneously, suggesting that YidC indeed functions as a monomer.

## Introduction

The plasma membranes of bacteria contain about a thousand different integral proteins with various topologies^1^. During their biosynthesis, most of these proteins insert into the membrane in an unfolded state by interaction with YidC or with the Sec translocase^2^. Under certain conditions, the Sec translocase can form a large complex consisting of SecYEG, and SecDF, YajC and YidC^3^. In this Sec holo-translocon, the peripheral SecA protein may participate using ATP hydrolysis to promote the translocation of large periplasmic substrate domains^4^.

The 10-spanning SecY protein of the SecYEG complex has an hour glass-like structure with a hydrophobic ring at its center that controls the passage of a protein chain through the membrane^5^. Membrane proteins are released from SecY through its lateral gate^6,7^. The membrane insertase YidC of *E. coli* is a 6-spanning membrane protein that is capable to catalyse membrane insertion of small proteins, e.g. the coat proteins of the filamentous phage M13 and Pf3^8–12^. In contrast to the Sec translocase, YidC does not possess a transmembrane channel. Instead, it has a hydrophilic groove in the inner leaflet of the bilayer and a transmembrane hydrophobic slide^13–15^. An open question is how a substrate protein interacts with YidC to achieve its integration into the membrane bilayer.

Whereas the molecular structure of both SecYEG and YidC show a single protein globule, they still may be present as oligomers in the membrane. Cryo-electron microscopy of SecYEG^16,17^ suggested a dimeric arrangement, possibly in a dynamic equilibrium with the monomer^18^. In addition, YidC and its mitochondrial homolog Oxa1 were found in dimeric complexes by cryo-electron microscopy^19^. Furthermore, Boy and Koch (2009) purified YidC from inner membrane vesicles as a 140 kDa protein and concluded that YidC appears mainly as a dimer in the cell^20^.

To test whether a dimeric YidC is functional and whether two copies functionally cooperate to insert a single substrate protein, we artificially constructed a YidC dimer by gene fusion. We show here that the YidC dimer is functional and can insert M13 procoat into the membrane. Also, the dimer is capable of inserting the C-tail protein SciP and the Pf3-Lep model protein that were previously characterized as YidC substrate proteins^21–23^. When one of the protomers is inactivated by a mutation, the dimeric YidC still retains activity, showing one active protomer is sufficient for YidC activity. Finally, we show that a YidC dimer can bind two substrate molecules simultaneously, suggesting that the functional unit of the insertase is a monomer.

## Results

### Artificial YidC dimer is functional as a membrane insertase

**Figure 1:**
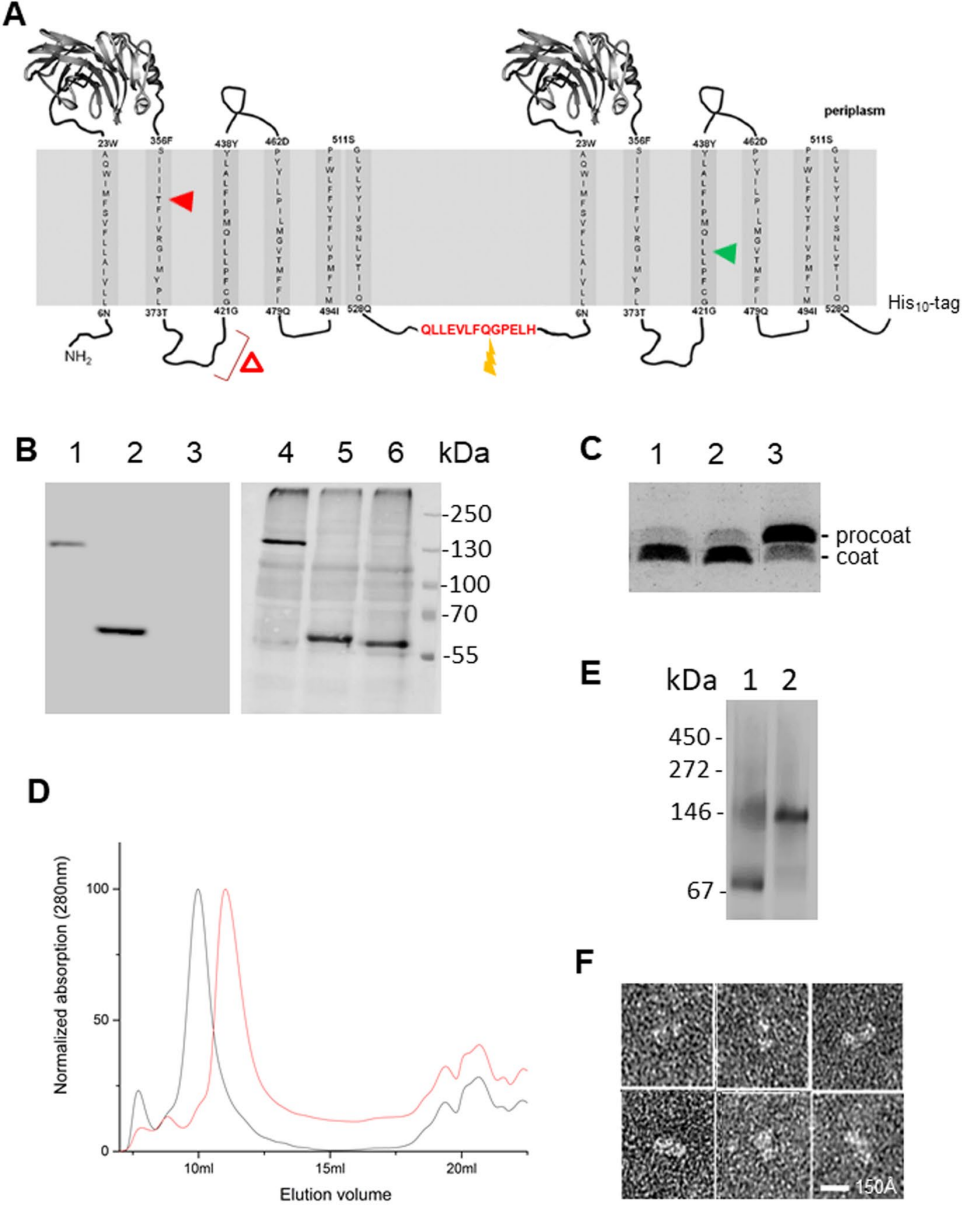
Construction, expression and activity of a dimeric YidC with the two protomers connected by a linker peptide. (**A**) Schematic and topology of a dimeric YidC. The linker between the protomers encodes a cleavage site for the prescission protease. The red arrowhead depicts the T362A mutation, the green arrowhead the position of the L427C used for crosslinking. The red triangle depicts the deletion of residues 399–415 in the ΔCH2 mutant. (**B**) Expression of the plasmid-borne cysteine-less dimeric YidC_0_/C_0_ (lane 1, 4), the monomeric YidC_0_ (lane 2, 5), and the wild-type YidC in non-depleted cells (lane 6) by Western blot with a his-tag antiserum (lanes 1–3) or with anti-YidC (lanes 4–6). The empty vector control is shown in lane 3. The cysteine at residue 423 of YidC was substituted with serine, designated as YidC_0_. (**C**) Cleavage of the M13 procoat protein to the mature coat by signal peptidase was monitored after membrane insertion by the dimeric YidC_0_/C_0_ (lane 1) or monomeric YidC_0_ (lane 2), respectively. In YidC-depleted conditions procoat cleavage was inhibited with the empty vector control (lane 3). (**D**) Size exclusion chromatography shows the profiles of purified monomeric (red line) YidC_0_ and dimeric (grey line) YidC_0_/C_0_, respectively. (**E**) Blue Native PAGE of inner membrane vesicles containing YidC_0_ (lane 1) or YidC_0_/C_0_ were analysed by Western blot according to Boy and Koch^20^. (**F**) Electron micrographs of purified YidC_0_/C_0_ after negative staining with uranyl acetate_._ Six particles were selected and they showed a size of approximately 10 × 20 nm.

To construct a dimeric YidC, the plasmid-encoded *yidC* gene was duplicated and the two copies of the gene were connected by a linker coding for a flexible 13 amino acid peptide (Fig. 1A). The activity of the YidC dimer was then tested after the plasmid was transformed into *E. coli* MK6 cells. In these cells the chromosomal *yidC* gene is under control of the *araBAD* promoter. Expression of YidC in the cells was analyzed in the absence of arabinose to deplete the chromosomally-encoded YidC and after induction of the *yidC* copy on the plasmid with IPTG (Fig. 1B). The cysteine-free protein dimer (designated YidC_0_/C_0_) showed the expected size of 120 kDa on a Western blot and no monomeric YidC was present (compare size with YidC_0_ control). A coexpressed M13 procoat protein was readily cleaved by signal peptidase (with its active site on the periplasmic side of the membrane) and hence inserted by either the monomeric YidC or the YidC dimer, respectively, proving that the dimer was a functional membrane insertase (Fig. 1C, lanes 1, 2). In contrast, when only the empty plasmid pGZ119HE was present uncleaved M13 procoat protein accumulated (lane 3).

In order to characterize the YidC dimer, the protein was expressed from *E. coli* MK6 cells and purified by NTA affinity chromatography, and its homogeneity analyzed by gel filtration (Fig. 1D). The dimeric protein eluted in earlier fractions than the monomer showing a single peak on the size exclusion column and a single band on SDS-PAGE. The size of the dimer on the column was determined to be roughly 200 KDa, corresponding to two YidC molecules in a detergent micelle. Blue-native PAGE of inner membrane vesicles from a cell lysate showed that YidC_0_/C_0_ (Fig. 1E, lane 2) comigrated with the dimeric YidC_0_ (lane 1). Also, the purified YidC dimer was examined by electron microscopy (Fig. 1F). The particles appeared as double-headed structures of the expected size.

### Both protomers of the YidC dimer are functional

**Figure 2:**
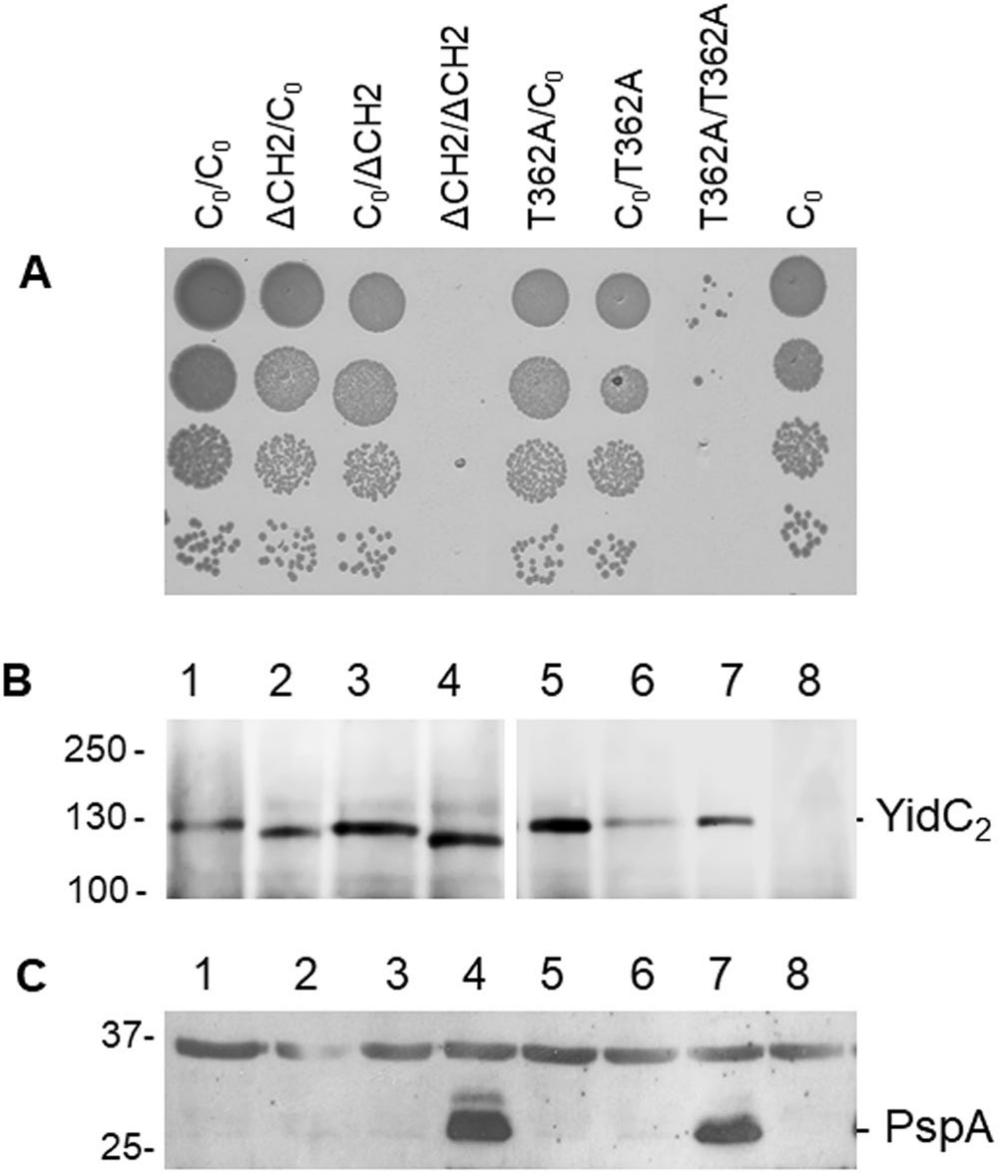
Complementation and expression of dimeric YidC mutants. The plasmid encoded YidC mutants were tested for growth in the YidC depletion strain MK6 with 0.4% glucose to deplete the chromosomally-encoded YidC. The genotype of each protomer is listed. C_0_ depicts the cysteine-less YidC, ΔCH2 the deletion of residues 399 to 415 in the C1 loop and T362A represents a single residue mutation in TM2. (**A**) When the cultures reached an OD_600_ = 0.5, serial dilutions were made and applied onto agar plates containing 0.2% glucose and 1 mM IPTG. (**B**) The same cultures expressing the dimeric YidC (as in A) were induced at OD_600_ = 0.5 for an additional 3 hours and the total cells were analysed by Western blot using His tag antibody. (**C**) Cultures expressing the various dimeric YidC constructs were grown under YidC depletion conditions for 5 h and analysed for induction of the phage shock protein PspA. The total cells were separated by SDS-PAGE and immunoblotted with PspA antiserum. Lane1 is the control with a cysteine-less YidC in both protomers (C_0_/C_0_), lane 2: ΔCH2/C_0_, lane 3: C_0_/ΔCH2, lane 4: ΔCH2/ΔCH2, lane 5: T362A/C_0_, lane 6: C_0_/T362A, lane 7: T362A/T362A, lane 8: YidC_0_.

To test which protomer contributes to the functional activity, either one of the two gene copies were replaced by a non-functional copy. As a non-functional YidC, we used two different mutants. First, a deletion mutant, YidC∆CH2, of the second helix in the first cytoplasmic loop region (amino acid residues 399 to 415^23^) and second, a mutation in residue 362 that changes the threonine into an alanine^24^ were investigated. The two YidC mutants did not complement the chromosomal depletion strain MK6 overnight using an agar plating assay (Fig. S1A). The MK6 cells were grown on plates with 0.2% glucose and 1 mM IPTG. This depletes the chromosomally encoded YidC and induces the expression of YidC mutants from the plasmid. Since *yidC* is an essential gene, colony formation is only observed when a functional version is expressed (Figs 2A, S1).

When both protomers had the ∆CH2 or the T362 A mutation, respectively, the dimers did not complement growth of the MK6 cells (Fig. 2A; see ∆CH2/∆CH2 or T362A/T362A mutants). The expression amount of the mutant proteins was tested after induction with 1 mM IPTG for 3 h and analysed by PAGE by Western blot (Fig. 2B). We used the induction of the phage shock response as an indicator of the activity of the mutated dimeric YidC insertases. Previously it was shown that an induction of the phage shock response is initiated when YidC function is inhibited likely to prevent membrane perturbation^25^ and that YidC complemented by a plasmid restores the Psp response to basal levels^26^. The readout of PspA expression measures the activity of YidC within the cell with low PspA expression conferring high YidC activity and high PspA expression conferring low YidC activity^26,27^. In order to monitor the induction of the phage shock response of the cells expressing YidC or the different mutants we examined the cellular levels of PspA by Western blot (Fig. 2C). While PspA expression was switched on in the cells with the double defective mutants, it is close to wild-type levels when one functional YidC protomer was expressed.

### Each protomer of the YidC dimer is functional as membrane insertase

When only one protomer had the ∆CH2 mutation, the YidC dimer was functional for complementation showing that one intact YidC protomer at either position is sufficient for activity (Fig. 2A). Similarly, a YidC T362A mutation was tolerated in either protomer. Taken together, these results suggest that membrane insertion requires only one functional protomer in the dimeric YidC. In most cases, this can be either the first or the second protomer.

**Figure 3:**
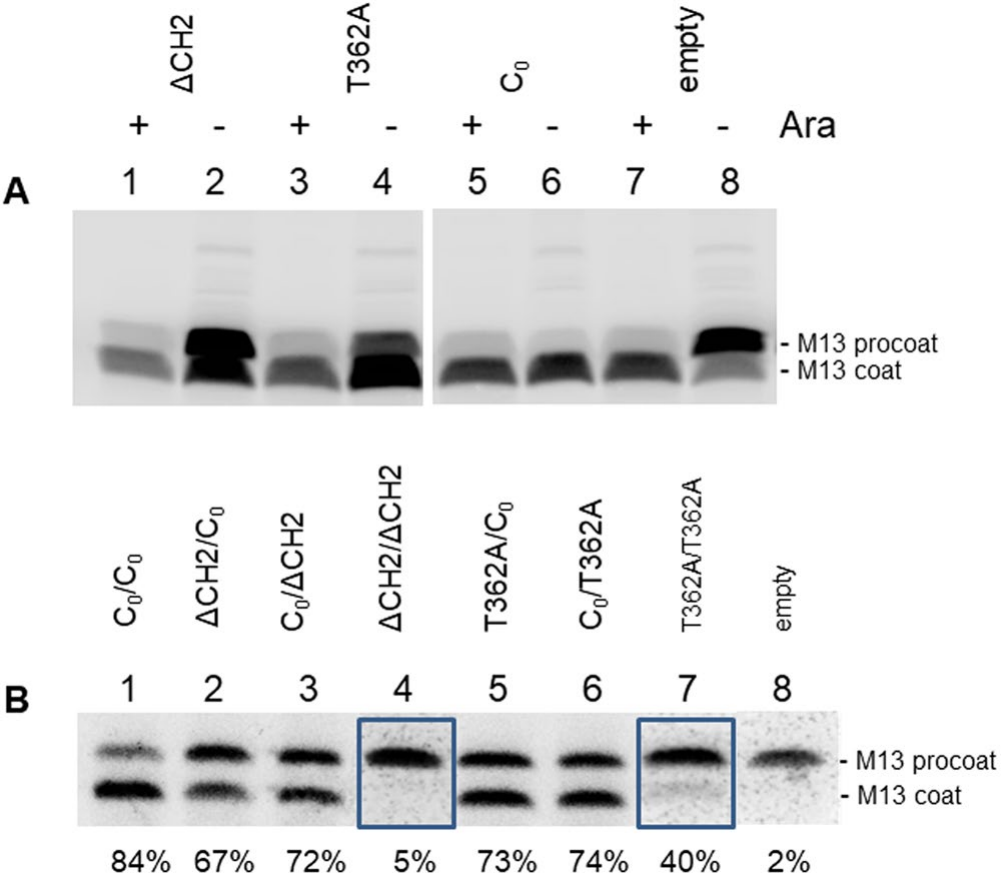
Each protomer of dimeric YidC is a functional membrane insertase for the M13 procoat. The membrane insertion of M13 procoat protein was monitored after pulse-labelling of MK6 cells expressing different YidC mutants. (**A**) MK6 cells expressing the monomeric YidC mutants (−Ara) with a deletion in the first cytoplasmic loop (ΔCH2, lanes 1, 2) and the point mutation T362A (lanes 3, 4), respectively, were analysed for M13 procoat cleavage. For a control, the chromosomal YidC was expressed (+Ara, odd numbered lanes). In addition, the amount of M13 procoat cleavage was monitored in the presence of the empty plasmid (lanes 7, 8) and compared to the plasmid-encoded YidC_0_ (lanes 5, 6). (**B**) MK6 cells expressing dimeric YidC with the mutations within the first, second or both protomers, respectively, were analysed for M13 procoat insertion and cleavage as described above. Lane1 is the control with a cysteine-less YidC in both protomers (C_0_/C_0_), lane 2: ΔCH2/C_0_, lane 3: C_0_/ΔCH2, lane 4: ΔCH2/ΔCH2, lane 5: T362A/C_0_, lane 6:C_0_/T362A, lane 7: T362A/T362A and lane 8: empty vector control. The boxed lanes (4, 7) with the double mutants were exposed for longer time (2×). Quantitation of membrane insertion of M13 procoat was performed as described in Methods.

The processing of the M13 procoat protein was taken as a measure of the activity of the insertase (Fig. 1C). Under depletion conditions most of the M13 procoat protein accumulates in its unprocessed form when the MK6 cells harbour an empty vector (Fig. 3A, lane 8). Mutants YidC ∆CH2 and T362A were inhibited in the processing of M13 procoat indicating that the mutations affect membrane insertion. All dimer YidC proteins with one functional protomer showed processing of M13 procoat (Fig. 3B) while when both protomers had the non-functional mutations (∆CH2/∆CH2 and T362A/T362A) they were blocked in membrane protein insertion. Interestingly, the insertase activity of each protomer contributed about equally (for example, compare lanes 5 and 6).

### The membrane insertion of SciP and Pf3-Lep is supported by one functional YidC protomer

**Figure 4:**
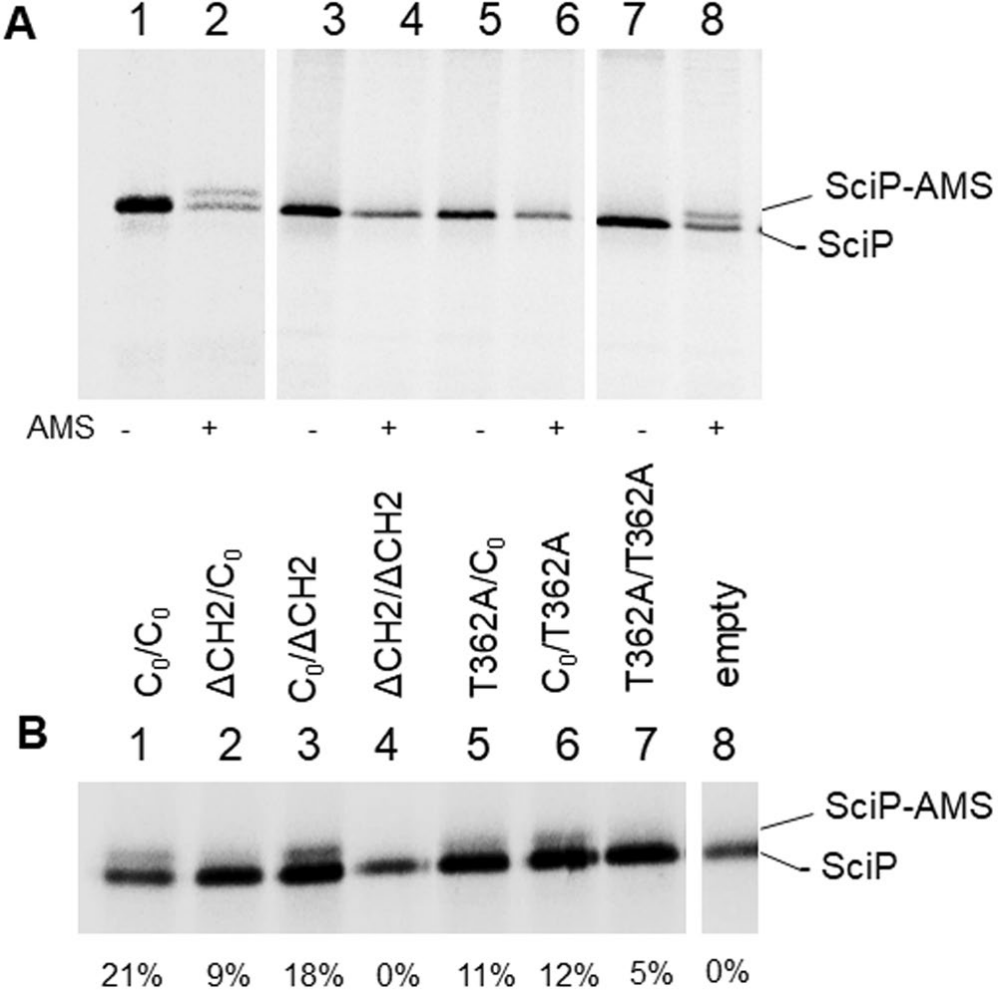
Insertion of SciP is affected by mutations in the YidC monomer and YidC dimer. (**A**) The insertion of SciP with a cysteine residue at 218 in the C-tail was expressed in MK6 (lanes 1, 2), coexpressed with YidC-ΔCH2 (lanes 3, 4), with YidC-T362A (lanes 5, 6) or with YidC_0_ (lanes 7, 8) were pulse-labelled for 3 min. The even-numbered samples were treated with AMS that shifts the protein by 0.5 kDa when the C-terminal tail was translocated to the periplasm. The total cell protein was immunoprecipitated with his tag antiserum and analyzed by SDS-PAGE and phosphorimaging. (**B**) Membrane insertion of SciP was monitored in cells coexpressing various YidC dimers as described above. Lane 1 is the control encoding a cysteine-less YidC in both protomers (C_0_/C_0_), lane 2: ΔCH2/C_0_, lane 3: C_0_/ΔCH2, lane 4: ΔCH2/ΔCH2, lane 5: T362A/C_0_, lane 6: C_0_/T362A, lane 7: T362A/T362A, lane 8: empty vector control. Quantitation was carried out as described in Methods.

As previously shown, YidC is required for insertion of the C-tailed membrane protein SciP^21,22^. The translocation of the small C-tail can be monitored by its modification with 4-acetoamido-4′-maleimidylstilbene-2,2′disodium sulfonate (AMS), where the periplasmic exposure of a cysteine results in an increased molecular weight of 0.5 kDa. The shift of the SciP protein was only observed for the wild-type (Fig. 4A, lane 2), whereas the ∆CH2 and T362A mutants did not show a shift of the SciP band (Fig. 4A, lanes 4 and 6) indicating that their membrane insertion was blocked. A similar outcome was observed with the dimeric YidC when both protomers had a mutated copy (Fig. 4B). The activity of the dimers with one functional protomer showed mostly a partial activity, similar to what was observed with the M13 procoat protein, described above. A position-dependent effect was observed for the ∆CH2 mutant as there was increased inhibition in membrane insertion when the mutation was in the first protomer.

**Figure 5:**
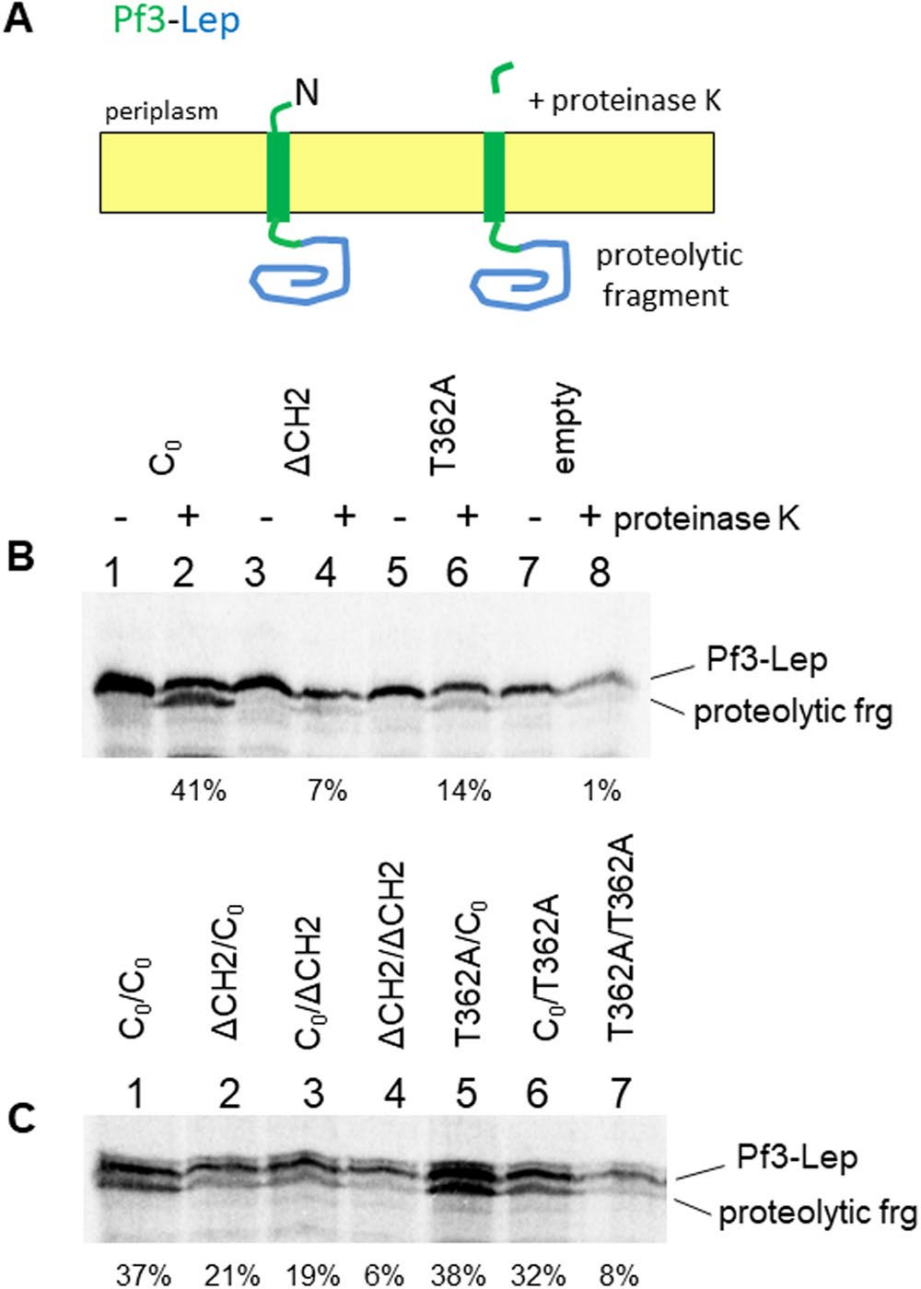
Both protomers of a dimeric YidC are active as a membrane insertase to integrate Pf3-Lep protein. (**A**) Schematic of transmembrane Pf3-Lep before and after proteinase K treatment. (**B**) The insertion of Pf3-Lep was coexpressed in MK6 with YidC-C_0_ (lanes 1, 2), YidC-ΔCH2 (lanes 3, 4), YidC-T362A (lanes 5, 6) or the empty plasmid (lanes 7, 8) and pulse-labelled for 1 min. The membrane insertion was assayed by proteinase K digestion when added to the periplasmic side of the cells. When the protein is membrane inserted the protease cleavage generates a slightly shifted fragment. The samples were immunoprecipitated with Lep antiserum and analyzed by SDS-PAGE and phosphorimaging. (**C**) Membrane insertion of Pf3-Lep was monitored in cells coexpressing various YidC dimers as described above. Lane1 is the control encoding a cysteine-less YidC in both protomers (C_0_/C_0_), lane 2: ΔCH2/C_0_, lane 3: C_0_/ΔCH2, lane 4: ΔCH2/ΔCH2, lane 5: T362A/C_0_, lane 6: C_0_/T362A, lane 7: T362A/T362A. Quantitation of translocation of N-tail of Pf3-Lep was performed as described in Methods.

Similarly, the Pf3-Lep fusion protein, which requires YidC for membrane insertion, was tested using the YidC dimer. We used protease accessibility of the N-terminal tail to monitor membrane insertion^23^. The proteinase K generates a proteolytic fragment that indicates the insertion of Pf3-Lep and that can be immunopreciptated with Lep antiserum (Fig. 5A,B). First, we confirmed for the monomeric YidC insertase mutants that the ∆CH2 and T362A mutations inhibited membrane insertion of Pf3-Lep, similar as was seen with the procoat and SciP substrates (Fig. 4A). When both protomers of the dimeric YidC contained the ∆CH2 and T362A mutations, membrane insertion was strongly inhibited while the YidC constructs retained activity when one functional protomer was present (Fig. 5C).

It should be noted that insertion of Pf3-Lep and SciP was not as efficient as previously seen^22,23^ since in the present study both the YidC mutant and the substrate protein are expressed on IPTG inducible plasmids for a short induction time while in the latter studies YidC was continuously expressed at the normal level.

### Mutations affecting one protomer are unable to suppress a mutation in the second protomer

**Figure 6:**
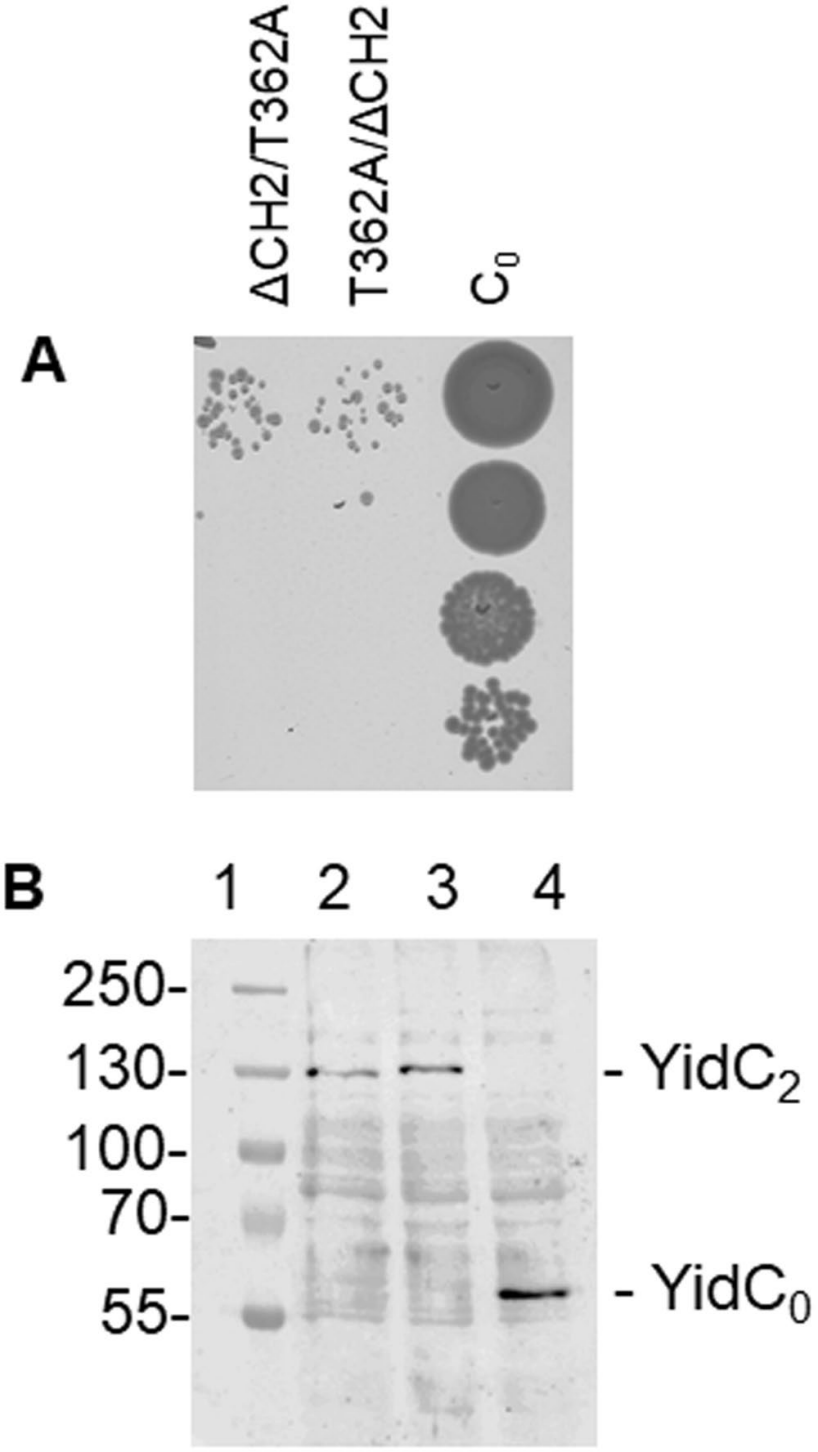
Activity and expression of dimeric YidC mutants with two defective protomers. Dimeric YidC mutants were constructed with a deletion within the C1 loop (residues 399 to 415) and a point mutation of T362A in either protomer. The mutant proteins were stably expressed as determined by Western blotting (**B**) but did not complement for growth in MK6 cells (**A**). For a control, the monomeric cysteine-less YidC (C_0_) was analysed.

Since one protomer was sufficient for YidC to function as a membrane insertase, we combined two different deficient protomers and investigated whether they are capable of intramolecular complementation. The deleted cytoplasmic C1 loop (ΔCH2) in one protomer was combined with a mutation of T362A expected to perturb the hydrophilic groove within the transmembrane domain^14^. Regardless of which protomer had either mutation both proteins were not functional in our *in vivo* complementation assay (Fig. 6A). In all cases, the dimeric YidC proteins, which were non-functional, were expressed at a normal level (see Fig. 6B).

### The YidC dimer binds two M13 procoat proteins

**Figure 7:**
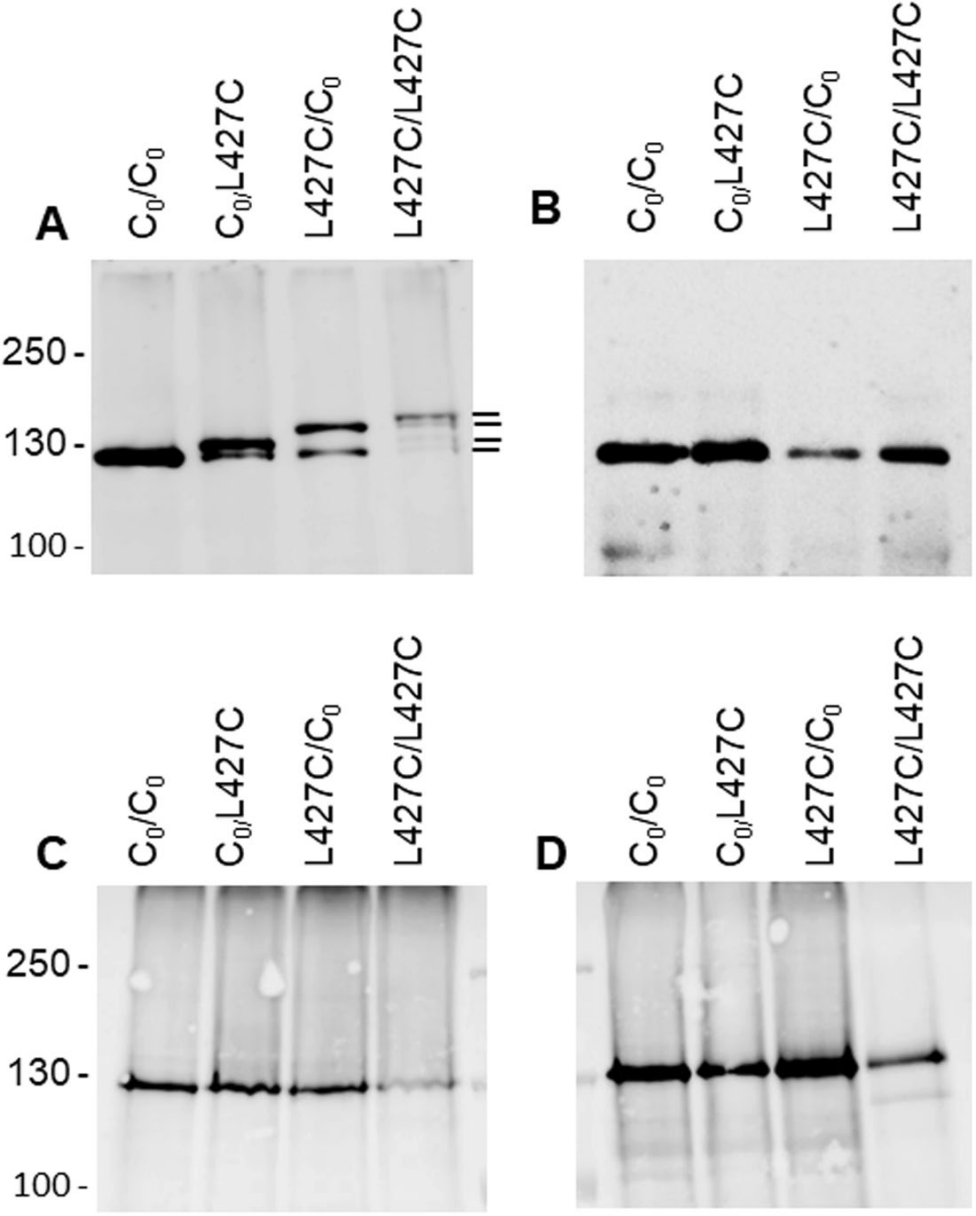
Dimeric YidC binds two M13 procoat proteins. (**A**) YidC cysteine-less dimer protein (lane 1), a mutant with the 427 C mutation in the second protomer (lane 2), in the first protomer (lane 3), or in both protomers (lane 4) were coexpressed with M13 procoat 33 C and crosslinked using DTNB for oxidation. The total cell protein was analysed on SDS-PAGE and the YidC protein bands were visualized on a Western blot. The YidC-procoat crosslinking leads to three different band shifts, depending on if the crosslink is to the second, first and both protomers, respectively. (**B**) As a control, the same samples as in (A) were analysed in the presence of DTT. (**C**) As in (A) but with a M13 cysteine mutant in the periplasmic loop region (+13 C) and (**D**) with cells not expressing M13 procoat. For all the panels, lane 1 is the control encoding a cysteine-less YidC in both protomers (C_0_/C_0_), lane 2: C_0_/427 C, lane 3: 427 C/C_0_, lane 4: 427 C/427 C.

The above complementation and activity results with a YidC dimer possessing one inactive protomer suggest that the two protomers operate on their own. However, it is still possible that two dimers come together to contribute their functional subunits to form a complex conferring insertase activity. To rule this latter possibility out, we examined substrate binding within the dimeric YidC. The contact site mutation 427 C was introduced into the first, the second or both protomers of YidC and coexpressed with M13 procoat 33 C (Fig. 7). When only the second protomer had the cysteine at position 427, a small shift was observed on the gel in comparison to the non-cysteine containing dimeric YidC (Fig. 7A, lane 2). The shift indicates a disulphide crosslink occurred between YidC and procoat, and is reversible when DTT was added (Fig. 7B). Interestingly, a bigger shift was obtained for the 427 C mutant in the first protomer (lane 3). This allows us to distinguish which of the two protomers has a substrate bound. For a double Cys dYidC with 427 C in both protomers, we observed the biggest shift with a size of about 20 kDa suggesting crosslinking of two procoat proteins to both YidC protomers, distinguishable from the weak bands corresponding to dYidCs with one procoat bound (lane 4). We conclude from this result that both protomers are functional and they each bind a substrate protein. For a control, a M13 procoat mutant with a cysteine in the periplasmic loop at position 13 was tested which was unable to crosslink with YidC-427C (Fig. 7C). Also, the cells not expressing the M13 procoat protein did not show crosslinking ruling out that another protein can crosslink to YidC-427C (Fig. 7D).

### Verification that each protomer binds one procoat protein after site-specific proteolysis

**Figure 8:**
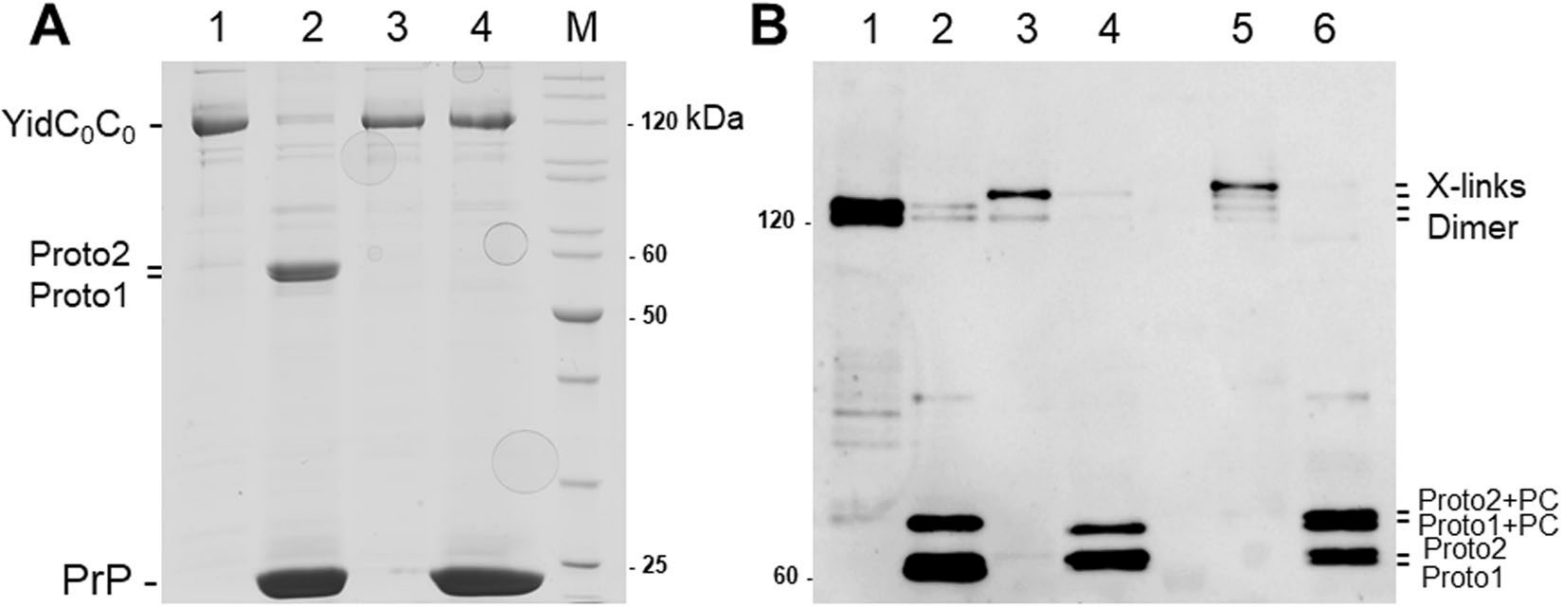
Each protomer of the dimeric YidC binds one M13 procoat protein. (**A**) The purified YidC dimer (lane 1) was incubated with prescission protease at 4 °C for 3 h. The two protomers were separated by SDS-PAGE (lane 2). Proto1 refers to the first protomer and Proto2 for the second encompassing the his-tag. For a control, the YidC dimer lacking the prescission recognition site (lane 3) was incubated with the protease (lane 4). The protein bands were visualized by Coomassie blue staining. (**B**) YidC dimer proteins with the 427 C mutation in the second protomer (lane 1, 2), or in the first protomer (lane 3, 4), or with cysteines in both protomers (lane 5, 6) were coexpressed with procoat 33 C, affinity purified and digested with the prescission protease (even numbered lanes). The protein bands were visualized by immunodetection with an anti-YidC antiserum.

To investigate the substrates that are crosslinked, the dYidC was purified and tested whether it has a substrate bound. Since the two YidC protomers are linked with a peptide encoding a recognition site for the prescission protease Prp, the purified dimer was cleaved after incubation for 3 h at 4 °C with the protease (Fig. 8A). After cleavage, the two protomers had a slightly different size resulting in a double band on PAGE (lane 2). This is explained by the fact that the C-terminal protomer has a 10 his tag, which increases its size on a SDS-PAGE gel. As expected, we observed no cleavage of the dimeric YidC, lacking the protease site for Prp, when the protease was added (lanes 3 and 4). When the YidC dimer had the 427 C mutation in the second protomer and was coexpressed with procoat 33 C the purified dimer shifted to a higher molecular weight indicating that it was crosslinked with the substrate (Fig. 8B, lane 1). Cleavage by prescission protease led to the separation of the two protomers visible as a doublet band at about 60 kDa (lane 2). The protomer with the crosslinked substrate generated a clearly shifted band. A similar result was obtained with the YidC dimer that had the 427 C mutation in the first protomer (lanes 3, 4). However, when the YidC dimer had the 427 C mutation in both protomers (lanes 5, 6), two shifted bands were observed after cleavage. This verifies that the dimeric YidC binds two substrate proteins, one in each of its protomers and the results are not consistent with two YidC protomers forming one binding site for a single substrate.

## Discussion

The oligomeric state of membrane proteins is often difficult to analyse since their affinity for each other and concentration in the membrane may be quite low. For the membrane insertase YidC, monomeric^13,14^ and dimeric forms^19,28^ have been isolated. In addition, YidC that is linked to SecYEGDFYajC has been documented[3]. These observations suggest a very dynamic behaviour for YidC in the plasma membrane of *E. coli* and lead to the question of which of these different forms are biologically active.

To investigate whether a dimeric YidC is functionally active and whether the two protomers are coordinately engaged to bind one substrate protein, we artificially linked two *yidC* genes on a plasmid with a short linker sequence between the two copies. Expression of the fusion protein produced a stable 120 kDa protein that appeared as a double-headed structure in the electron microscope (Fig. 1F). The expression of the YidC dimer in *E. coli* MK6 cells under conditions where the chromosomally-encoded copy is repressed showed that it fully complements colony growth on agar plates. Likewise, the conditions where only the dimeric YidC is expressed showed that M13 procoat protein is membrane inserted and cleaved to coat by signal peptidase on the *trans* side of the membrane, corroborating that the dimeric YidC is functional as an insertase.

Do the two protomers of the YidC dimer act independently as two insertases or as one unit for protein insertion? When defective mutations were incorporated into one of the two protomers, the dimeric protein complemented the YidC depletion strain and the phage shock response was not switched on. Since switching on the phage shock response is an indicator of the respiratory subunits not being inserted^27,29^, we infer from this result that these subunits are being inserted by the dimeric protein with one defective protomer but not when both protomers were defective (Fig. 2C). Additionally, these YidC insertases with one functional protomer still retained the ability to insert the M13 procoat, SciP or Pf3-Lep. This was observed for a YidC mutant with a mutation in the C1 domain (ΔCH2) suggesting that each C1 domain acts by itself and does not require a dimeric state. Similarly, the most effective point mutation, T362A, for inhibition of YidC still allowed membrane insertion of all 3 substrate proteins when only one YidC protomer had this mutation. Since the residue 362 is expected to disrupt the hydrophilic groove of YidC^14^, these results are consistent with a single hydrophilic groove being functional. It is therefore unlikely that the two YidC protomers must form a common groove in order for insertase to function.

We rationalized that if the two protomers operate independently they also should then each bind to a substrate protein. This was tested by introducing a cysteine at position 427 in TM3, either in one or both protomers of the YidC dimer and performing disulphide crosslinking with a co-expressed M13 procoat substrate. The crosslink of YidC 427 C with M13 procoat + 33 C resulted in a shift on SDS-PAGE (Fig. 7) and the shift was more pronounced if the 427 C was in the first protomer compared to when it was in the second protomer. Importantly, when both protomers possessed a cysteine mutation, the shift was even larger (lane 4) suggesting that two M13 procoat molecules were bound to the YidC dimer. This was corroborated by cleaving the two protomers in the linker region with the prescission protease (Fig. 8). After cleavage, both protomers possessing a cysteine showed a shifted band suggesting that each protomer has a crosslinked M13 procoat protein.

Although the two protomers in the dimeric YidC are both functional as independent insertases they seem to show some different features. For example, the substrate binding as determined by disulphide crosslinking affects the migration of the dimeric YidC differently. We found the covalently-bound substrate to the first protomer leads to a substantial higher shift on SDS-PAGE than binding to the second protomer in the YidC dimer. However, when the dimer was cleaved by the prescission protease the shift of both protomers was almost identical except for the small difference in size due to the His tag on the second protomer (Fig. 8B, lane 6). Hence, the protomer position in the dimer appears non-symmetric. A slight position-dependent effect was also observed for the ∆CH2 mutant (Fig. 4B). We think that steric reasons can explain the differences of the two linked insertases. Possibly, the substrate accessibility or the SecY interaction with the second protomer might be not as efficient as for the first protomer which is freely exposed at the N terminus.

In conclusion, the YidC dimer contains two functional protomers although they can have some restrictive influence on each other. Each protomer is capable to bind one substrate protein and catalyses its membrane insertion.

## Methods

### Bacterial strains and plasmids

YidC depletion was carried out using *E. coli* strain MK6^30^, a strain where the promoter of the *yidC* gene had been exchanged with the *araC-araBAD* promoter cassette. Depletion of the chromosomally encoded YidC was achieved by growth of MK6 in the presence of 0.4% glucose. For complementation assays, MK6 was transformed with pGZ119EH^31^ expressing the respective YidC proteins under the control of the *tac* promoter. Coexpression of M13 procoat was performed with pMS-M13procoat^32^ and Sci-P with pMS-SciP-C^22^. Where indicated, the M13 procoat wildtype and H5-33C mutant were expressed from the pMS119EH vector.

### Genetic construction of the dYidC

Plasmid pGZ119-YidC_0_^30^ was used as a template for cloning. The *yidC* gene was amplified from the plasmid with two oligonucleotides adding sites for XbaI and SphI in front of the gene and a MfeI site at the end of the gene. This construct was then digested with XbaI and SphI, and the fragment with the *yidC* was subcloned into pGZ119 YidC_0_ plasmid, resulting in pGZ119 dYidC_0_/C_0_. Between the two *yidC* genes a linker sequence was inserted into the MfeI site encoding the recognition sequence for the prescission protease. In all experiments, except when mentioned otherwise, the version with the prescission linker was used.

The various YidC mutants with changes in the first or the second copy were constructed by site directed mutagenesis on the plasmid which encodes either the first or the second YidC copy. After mutagenesis, the mutated copy was again subcloned as N-terminal or C-terminal protomers, or in both protomers to obtain pGZ119 dYidC.

### *In vivo* complementation assay

YidC complementation was carried out using *E. coli* strain MK6^30^ harbouring the respective YidC mutants on the pGZ119-YidC plasmid. Overnight cultures in LB medium, containing 25 µg/mL chloramphenicol, 0.2% arabinose and 0.4% glucose were washed with LB and inoculated 1:100 into fresh LB medium with 25 µg/mL chloramphenicol and 0.4% glucose to deplete the chromosomal YidC. After growth to OD_600_ = 0.5 a serial dilution in LB medium (no additives) was prepared from 10^−1^ to 10^−5^. The dilutions were spotted (3 µL) on LB agar plates containing 0.2% arabinose, 0.2% glucose and, when indicated, 1 mM IPTG for induction of the mutants. The agar plates were incubated at 37 °C overnight.

### Expression of the YidC and dYidC mutants

To control expression of YidC and dYidC, the above mentioned cultures at OD_600_ = 0.5 were induced with 1 mM IPTG for 3 h. The cultures were then harvested and had an OD_600_ = 1.63 ± 0.06. The cells were precipitated with a final volume of 20% TCA at 4 °C overnight, centrifuged and washed with acetone. The dried cell pellet was solubilized in gel loading buffer and analyzed on a 10% SDS-gel followed by Western blotting and immunodetection with a His tag antibody.

### Detection of the phage shock protein A

Overnight culture of MK6 cells harbouring various dimeric YidC constructs were washed twice with LB medium and inoculated 1:100 into fresh LB medium with 25 µg/mL chloramphenicol, 50 µM IPTG and 0.2% glucose to deplete the chromosomal YidC. After 5 h of depletion, cell density was adjusted to OD_600_ = 0.4 in all cases. 500 μL of cells were mixed with equal volume of 20% TCA, incubated on ice for 1 h, washed with acetone, and solubilized in Tris-SDS buffer. The sample was then added directly to SDS-PAGE gel loading buffer and PspA was detected by immunobloting using HyGLO^TM^ chemiluminescent detection kit (Denville Sci. Inc.).

### Translocation assay with the wild-type M13 procoat

The wild-type M13 procoat expressed from a pMS119EH derivative was co-expressed with one of the various defective YidC mutants to test for their capability of inserting a substrate. Overnight cultures of MK6 harboring two plasmids, one encoding the YidC defective mutants or the YidC controls (pGZ119EH) and the other the wild-type M13 procoat (pMS119EH), were grown in LB (200 µg/mL ampicillin, 25 µg/mL chloramphenicol, 0.2% arabinose, 0.4% glucose) overnight at 37 °C. The cells were washed twice with LB medium and then inoculated 1:100 in fresh LB medium (200 µg/mL ampicillin, 25 µg/mL chloramphenicol, 0.4% glucose) and grown under YidC depletion conditions to an OD_600_ = 0.5 at 37 °C. 200 µL were taken and washed twice and resuspended in 200 µL M9 medium lacking methionine. After 45 min growth at 37 °C 1 mM IPTG was added for 10 min. 20 µCi [^35^S]-methionine was added for 1 min, followed by the addition of 10% TCA.

### Immunoprecipitation

The TCA precipitated cells were washed with acetone and centrifuged for 5 min at 4 °C. After drying at 95 °C for 5 min, 50 µL of resuspension buffer (2% SDS, 10 mM Tris, pH 8) was added and the pellet was incubated at 95 °C for 5 min. 1 mL of TEN-TX buffer (150 mM NaCl, 10 mM Tris, 1 mM EDTA, 2% Triton X-100, pH 8) and 20 µL of Staph-A (Zymed) were added and incubated at 4 °C on a rotating wheel for 30 min. The samples were then centrifuged for 5 min at 4 °C to pellet the Staph-A. The supernatant was transferred to a new tube containing His tag (or M13) antibody for immunoprecipitation. The samples were incubated on the rotation wheel overnight at 4 °C before adding 20 µL of Staph-A for 1 h. Staph-A was pelleted by centrifugation for 1 min at 4 °C, washed twice with TEN-TX buffer and once with TEN-buffer (150 mM NaCl, 10 mM Tris, 1 mM EDTA, pH 8). The pellets were resuspended in 50 µL gel loading buffer with or without 100 mM DTT and heated at 95 °C for 5 min. The samples were centrifuged for 2 min and the supernatant loaded on a SDS polyacrylamide gel without disturbing the Staph-A pellet.

### Translocation assay of SciP with AMS

The plasmid-encoded SciP-C^22^ was co-expressed with one of the various defective YidC mutants in MK6 cells in LB (100 µg/mL ampicillin, 37 µg/ml chloramphenicol, 0.4% glucose) under YidC depletion conditions to an OD_600_ = 0.5 at 37 °C. After resuspension in M9 medium lacking methionine the cells were grown for another 45 min, induced with 1 mM IPTG for 10 min. 2.5 mM AMS (Molecular Probes) was added for 1 min and then labeled with 15 µCi ^35^S-methionine for 2 min. Nonradioactive L-methionine was added for 10 min, and the AMS reaction was quenched by the addition of 10 mM DTT for further 10 min. The cells were acid-precipitated and immunoprecipitated (his tag antibody) as previously described.

### Membrane translocation of the N-terminal tail of Pf3-Lep

pMS119 bearing Pf3-Lep was expressed with one of the YidC constructs to examine N-tail translocation. Overnight cultures of the YidC depletion strain MK6 bearing two plasmids, one encoding the YidC defective mutant or the YidC blank control (pGZ119EH) and the other Pf3-Lep (pMS119), were grown in LB (50 μg/mL ampicillin, 25 μg/mL chloramphenicol, 0.2% arabinose) overnight at 37 °C. After washing the cells twice in LB medium, the cells were inoculated 1:100 in fresh LB medium (50 µg/mL ampicillin, 25 µg/mL chloramphenicol, 0.2% glucose) and grown under YidC depletion conditions for 6 h. In the last 10 min of the 6 h period, 50 µM IPTG was added to induce the expression of YidC defective mutant. The cells were then washed with M9 medium and transferred into M9 lacking methionine, and grown for 30 min. The YidC construct and Pf3-Lep was induced with 1 mM IPTG for 2 min, and then pulse-labeled with [^35^S]-methionine for 1 min. Cells were collected by pelleting in a microfuge at 4 °C and resuspended in 40% sucrose and 33 mM Tris-HCl (pH 8.0). Cells were converted into spheroplasts by treatment with lysozyme (5 µg/mL) and 1 mM EDTA (pH 8.0) on ice for 30 min. The spheroplasts were treated with or without proteinase K (0.8 mg/mL final concentration) for 1 h on ice. 5 mM PMSF (final concentration) was added to stop the protease reaction. Pf3-Lep was immunoprecipitated with leader peptidase antibody and samples analyzed by SDS-PAGE and phosphorimaging.

### Quantitation of translocation of the polar region of Procoat- and Pf3-Lep

The band intensity (without gel background) of the inserted protein was quantified using the phosphorimaging Aida Analyzer (Raytest, Straubenhardt). For quantification of the membrane insertion of M13 coat protein we took into consideration that the membrane inserted and processed coat has only one methionine whereas the non-processed procoat has 3 methionines. For Pf3-Lep, the proteolytic fragment has 6 methionines, whereas the intact Pf3-Lep has 7.

### Purification of cysteine-less YidC and dYidC

For purification, tryptone medium was used to grow the MK6 cells bearing pGZ119-dYidC. 0.5 mM IPTG was added at an OD_600_ = 0.7 and growth of the culture was continued for 4.5 h. The cultures were then harvested and resuspended in buffer A (10% sucrose, 50 mM Tris-HCl, pH8, 500 mM NaCl, 1 mM EDTA) followed by disruption with a One Shot cell disruption system (Constant Systems LTD). The lysed cells were centrifuged (8,000 × g for 10 min at 4 °C) to precipitate cell debris. The supernatant was transferred to an ultracentrifuge tube and the membrane vesicles spun down at 140,000 × g for 2 h at 4 °C. The membrane pellet was resuspended in buffer A and loaded on a three step sucrose gradient (25%, 50%, 70%) in 50 mM Tris-HCl, pH 8, 1 mM EDTA to separate the inner and outer membrane vesicles. The resuspended membrane vesicles were loaded on the gradient and centrifuged at 110,000 × g for 16 h. The inner membrane vesicles (IMVs) concentrate at the separation layer between sucrose step 1 and 2 while the outer membrane vesicles (OMVs) are between step 2 and 3. The IMVs were carefully collected with a syringe and mixed 1:4 with sucrose-free Tris buffer (50 mM Tris-HCl, pH 8). The IMVs were then centrifuged at 140,000 × g for 2 h at 4 °C and resuspended in buffer B (10% glycerol, 50 mM Tris-HCl, pH 8, 500 mM NaCl). IMVs were solubilized with 2% DDM for 2 h and centrifuged at 100,000 × g for 1 h at 4 °C. YidC in the supernatant was then purified by immobilized metal ion chromatography (IMAC). Ni-Sepharose beads were prepared by washing twice with 10 column volumes (CV) with buffer C (10% glycerol, 50 mM Tris-HCl, pH 8, 500 mM NaCl, 20 mM imidazole, 0.03% DDM). 20 mM of imidazole was also added to the solubilized membranes before adding the Ni-Sepharose. After 2 h of incubation on a rotary wheel at 4 °C, the column was run by gravity flow, washed with 40 CV of buffer D (10% glycerol, 50 mM Tris-HCl, pH8, 500 mM NaCl, 40 mM imidazole, 0.03% DDM) and then eluted with the buffer E (10% glycerol, 25 mM MES, pH 6.5, 500 mM NaCl, 600 mM imidazole). The protein was further purified by size exclusion chromatography using a Superdex 200 10/300 at RT in 10% glycerol, 10 mM Ada (acetamidoimino-diacetic acid), pH 6, 300 mM NaCl, 0.02% DDM, 0.01% Cymal6). The collected fractions were analyzed by SDS-PAGE and visualized by Coomassie staining.

### Purification and cleavage of dYidC by prescission protease

For testing the accessibility of dYidC to the prescission protease, two versions (one with and one without the prescission protease site) of the YidC dimer were purified as described above, except the sucrose centrifugation and gel filtration step. Prescission protease was added to the purified YidC in 300 mM NaCl, 50 mM Tris-HCl, pH 8, 1 mM EDTA and incubated at 4 °C for 3 h prior to terminating the reaction by the addition of 75% methanol.

### Substrate crosslinking with dYidC

Overnight MK6 cultures in LB (200 µg/ml ampicillin, 25 µg/ml chloramphenicol, 0.2% arabinose, 0.4% glucose), harboring plasmids encoding cysteine mutants of YidC (pGZ119EH) and M13-H5+33C procoat (pMS119EH) were inoculated 1:100 into fresh LB medium and grown under depletion conditions to an OD_600_ = 0.5 at 37 °C. 1 mM IPTG was added for 10 min, followed by the addition of 200 µM DTNB for 3 h. Then, 20% TCA was added to the cultures and stored overnight at 4 °C followed by processing for PAGE and immunodetection (his tag antibody). For cleavage with prescission protease of the dYidC-substrate complexes, the proteins were purified and cleaved as described above and subsequently analyzed via PAGE and immunodetection.

## Electronic supplementary material

Dataset 1
