# Supplementary material for: Each protomer of a dimeric YidC functions as a single membrane insertase
Source: Sci Rep. 2018 Jan 12;8:589. doi: 10.1038/s41598-017-18830-9 (PMC5766580; doi:10.1038/s41598-017-18830-9)
Supplement: Supplementary file 1 — Dataset 1 [file 41598_2017_18830_MOESM1_ESM.rtf]

Each protomer of a dimeric YidC functions as a single membrane insertase


Dirk Spann1, Eva Pross1, Yuanyuan Chen2, Ross E. Dalbey2
and Andreas Kuhn1*


C0/C0

ÄCH2/C0

C0/ÄCH2

ÄCH2/ÄCH2

T362A/C0
C0/T362A T362A/T362A C0


C0/C0

ÄCH2/C0

C0/ÄCH2

ÄCH2/ÄCH2

T362A/C0
C0/T362A T362A/T362A C0


C0

ÄCH2

T362A


C0

ÄCH2

T362A


C0

ÄCH2

T362A


Figure S1


(A) Complementation of monomeric YidC mutants. The plasmid encoded YidC mutants were tested for growth in the YidC depletion strain MK6. The genotype of each protomer is listed. C0  depicts the cysteine-less YidC, ÄCH2 the deletion of residues 399 to 415 in the C1 loop and T362A a single residue mutation. Serial dilutions of cells bearing the respective mutant on agar plates with 0.2% arabinose (left panel), 0.2% glucose (middle panel) and 0.2% glucose with 1 mM IPTG (right panel).


(B) Complementation  of  dimeric  YidC  mutants.  Controls  of  the data presented in Fig. 3 showing the agar plates with 0.2% arabinose (left panel) and 0.2% glucose (right panel).
